# Metabolic Hormone Profiles in Breast Milk From Mothers of Moderate-Late Preterm Infants Are Associated With Growth From Birth to 4 Months in a Sex-Specific Manner

**DOI:** 10.3389/fnut.2021.641227

**Published:** 2021-05-28

**Authors:** Laura Galante, Clare M. Reynolds, Amber M. Milan, Tanith Alexander, Frank H. Bloomfield, Yannan Jiang, Sharin Asadi, Mariana Muelbert, David Cameron-Smith, Shikha Pundir, Mark H. Vickers, the DIAMOND study team

**Affiliations:** ^1^The Liggins Institute, University of Auckland, Auckland, New Zealand; ^2^School of Public Health, Physiotherapy and Sports Science, Institute of Food and Health, Conway Institute, University College Dublin, Belfield, Ireland; ^3^Food Nutrition & Health Team, Food & Bio-Based Products Group, AgResearch, Palmerston North, New Zealand; ^4^Neonatal Unit, Kidz First, Middlemore Hospital, Auckland, New Zealand; ^5^Department of Statistics, Faculty of Science, University of Auckland, Auckland, New Zealand; ^6^Singapore Institute for Clinical Sciences, Agency for Science, Technology and Research, Singapore, Singapore

**Keywords:** adiponectin, breastmilk, breastfeeding, human milk, IGF-1, growth trajectories, leptin, pre-term birth

## Abstract

**Background:** Differing environmental conditions experienced by mother-infant dyads may influence composition of the milk received by the infant. As a consequence, diverse milk compositional profiles may contribute to different postnatal outcomes, especially in infants facing adverse perinatal environments. We investigated whether variability in milk concentrations of key metabolic hormones is associated with different growth outcomes in infants born preterm, a perinatal complication known to impact on infant growth.

**Methods:** Human milk samples were collected from 169 mothers of 191 infants enrolled in the DIAMOND trial, a randomized trial of nutrition for moderate-late preterm infants, at 5 and 10 days postpartum and again at 4 months' corrected age and analyzed for leptin, adiponectin and insulin-like growth factor (IGF)-1. Infant weight and body composition were measured at birth, discharge and 4 months' corrected age. Multiple linear regression models were used to examine correlations between milk hormone concentrations, weight z-scores and body composition at discharge and 4 months' corrected age, and weight gain from birth to 4 months' corrected age. Sex-specific interactions were examined.

**Results:** Higher milk IGF-1 concentrations on day 5 after birth were associated with greater infant fat-free mass at 4 months' corrected age. Milk IGF-1 concentrations at 4 months were positively associated with fat mass and fat-free mass at 4 months in boys but not girls. Milk leptin concentrations on day 5 after birth were positively associated with fat mass at discharge from hospital, but negatively associated with fat mass at 4 months' corrected age. No significant association was found for milk adiponectin concentrations.

**Conclusion:** Milk IGF-1 and leptin concentrations in mothers of moderate-late preterm babies are associated with different growth and body composition through to 4 months' corrected age and these associations are often different in boys and girls. The sex-specific effects of nutrient and hormone exposure during early life in preterm infants warrants further investigation to optimize the nutritional care these infants receive, particularly in hospital, where the same nutrition is provided to boys and girls.

## Introduction

An increasing body of research has highlighted that the differential compositional nature of human milk (HM) reflects a complex interplay between maternal and infant characteristics as well as that of the surrounding environment (1–5). This makes HM the most dynamic, multifaceted and personalized nutrition that individuals consume in their lifetime and ultimately the best source of nutrition for human infants. Yet, lactation and milk composition are the result of millions of years of evolution, where specific selective pressures have shaped the breastfeeding process as a compromise between maternal and offspring health (6, 7). Energy requirements to breastfeed an infant are high for mothers (8) with studies in animal models showing that the nursing behavior of mothers changes drastically in response to low food availability. In this context, mothers have been shown to prioritize their own health and future reproductive potential over current reproduction (9, 10).

Clinical studies show that mothers of low socioeconomic status breastfeed less, suggesting that, in humans, breastfeeding itself can reflect a health behavior dictated by alterations in social conditions (11). In this context, if breastfeeding behavior can reflect specific environmental conditions, then it is also possible that HM composition may be affected by the circumstances faced by individual mothers. Independent of the associations between maternal phenotypic variability and HM composition, each mother-infant dyad is also exposed to a different environment and accordingly experiences different burdens (e.g., socioeconomic adversities, poor nutrition, lack of support within the family, and/or the work environment). Such environmental burdens may contribute, in part, to the selective pressure that determines what kind of subconscious biological compromises (i.e., trade-offs) each mother makes in order to balance their health and well-being with that of their infant. These trade-offs could therefore be reflected in HM composition, feeding decisions and ultimately influence postnatal outcomes (6, 12).

We have previously reported that perinatal health and environmental conditions including gestational diabetes (GDM), non-elective C-section, perinatal stress, and low maternal socioeconomic status are associated with variations in the concentration of HM metabolic hormones (4, 13). Such metabolic hormones contained in HM (i.e., leptin, adiponectin, and growth factors) regulate multiple biological functions (14) and are potential mediators of infant health outcomes across the life course by providing important environmental cues at critical time-points of developmental plasticity (15–17). In this context, our study in a healthy Finnish cohort has recently demonstrated that growth trajectories up to 5 years were associated with HM concentrations of insulin-like growth factor (IGF)-1 (18). Similarly, others have reported associations between HM composition and diverse infant growth trajectories (19–22). However, studies on this topic are to date scarce, often performed in very different settings, analysing different aspects of HM composition and not always pointing in the same direction.

An important aspect seldom taken into account is that associations between HM composition and infant outcomes may be particularly evident in mother-infant dyads that face health-related hardships in the perinatal period, including pre-term birth, which may coexist with social/environmental burdens, as suggested by recent research on the reduction of preterm birth during the COVID-19 lockdown (23). According to the Trivers-Willard hypothesis (24), sick or economically burdened mothers tend to produce more female than male offspring and vice-versa. While this hypothesis seems to have limited applicability in human populations, it is nonetheless supported by evidence suggesting that investing in sons is more costly for mothers than investing in daughters (25) and the phenomenon has also been observed in some human populations, with wealthier families producing more sons than daughters (26). This implies one or more mechanisms of sexual selection that, based on environmental conditions determines the sex ratios of newborns. In humans, the birth sex ratio of newborns is typically shifted toward males, but at the same time more males are also born preterm (27) and are likely to suffer from postnatal morbidity and mortality (28, 29). While the causes of the human sex ratio at birth are complex and still unclear, it is possible that the differential perinatal mortality between males and females might be part of a sexual selection mechanism that favors one or the other sex depending on environmental conditions (30).

In this context, breastfeeding and HM are a form of parental investment and variations in either frequency of breastfeeding or compositional profile of HM seems to be linked to environmental conditions and are likely to affect postnatal outcomes in a sex-specific manner. While differences in the composition of HM according to the sex of the infant have previously been reported, the composition of HM, particularly in terms of metabolic hormones, has not been comprehensively assessed in relation to sex-specific postnatal outcomes in preterm infants (31). We therefore aimed to investigate infant growth outcomes in relation to HM metabolic hormone variability in a subset of infants enrolled in the DIAMOND trial, a factorial randomized trial of different nutritional management in moderate-late preterm babies (32). We hypothesized that maternal own milk (MOM) metabolic hormone concentrations are associated with infant growth and body composition at discharge from hospital and at 4 months' corrected age and that infants of different sex have differential growth trajectories in association with similar concentrations of metabolic hormones in MOM.

## Materials and Methods

### Study Design and Population Characteristics

Participants are 169 mothers and 191 moderate-late preterm infants (32^+0^ to 35^+6^ weeks' gestation) enrolled in the DIAMOND trial (ACTRN12616001199404) (32) whose mothers consented to provide HM samples as detailed previously (33). Briefly, between May 2017 and June 2019, infants born between 32^+0^ and 35^+6^ weeks of gestation were recruited at four neonatal care units in Auckland, New Zealand (*n* = 225) and randomized within 24 h of birth to one of three nutrition interventions in a factorial trial design. Written informed consent was obtained from the parents for the enrolment of their infants. The study protocol was approved by the New Zealand Health and Disability Ethics Committee (16/NTA/90). Samples were collected on day 5 and 10 postpartum from mothers whose infants were still in hospital and at 4 months' corrected age from mothers who were breastfeeding and attended a follow-up appointment. MOM was collected preferably in the morning (10 a.m. −12 p.m.), 2–3 h after the previous feed and exclusively from the right breast, with the use of a hospital grade breast-pump (Medela, Baar, Switzerland). Infant anthropometry (weight, length, and head circumference) was measured at birth, discharge from hospital and at the 4-month visit. Z-scores were calculated based on the Fenton preterm growth charts (34) for the in-hospital measurement and on the World Health Organization (WHO) growth chart for the 4 month measurements (35) as this is a standard approach used for preterm cohorts (36). Body composition (fat mass and fat-free mass expressed in kg) was measured where possible at discharge and 4 months' corrected age by using air displacement plethysmography (PEA POD®, COSMED, Concord, CA, USA). In this instance, the protocol requires that infants are tested naked and with their hair flattened against their head with oil or a head cap. The PEA POD is calibrated for each scan and given that infant movement can impact on the measures taken, if 3 movement errors are detected by the device, all measures were automatically repeated. Two of the four recruiting units did not have access to a PEA POD machine. In addition, body composition data was not available for some infants due to an inability to attend a hospital or on-site follow-up visit, particularly in those in lower SES groups. In these cases, follow-up visits were conducted at the participant's home and thus body composition measurements were not available for these infants. Further, given the PEA POD device parameters (weight range 1–8 kg), there were a small number of infants who were too large for the device and thus did not have measurements taken at the time of FU. Waist circumference and mid-upper-arm circumference (cm) at 4 months' corrected age were also taken to use as a substitute for body composition in infants without PEA POD data. However, at present there is no published equation that is suitable for accurate use in this population (37). The current study utilized International Standards for Anthropometric Assessments published by the International Society for the Advancement of Kinanthropometry (ISAK) (38). Written informed consent was obtained from the parents for the enrolment of their infants. The study protocol was approved by the New Zealand Health and Disability Ethics Committee (16/NTA/90).

### MOM Sample Collection and Analysis

MOM samples were collected as specified previously (13). Analysis, validation and the analytical detailed process have been previously described (4). In brief, leptin, adiponectin and IGF-1 in MOM were analyzed using commercially available ELISA kits (human sensitive Leptin ELISA, human Adiponectin ELISA, human IGF-1 ELISA, Mediagnost, Reutlingen, Germany) and MOM total protein was quantified by infrared spectrometry in order to normalize hormone concentrations, using the Direct Detect® technology (Merck, Germany). The intra- and inter-assay coefficients of variation, respectively, for the ELISAs (QCs supplied) were adiponectin (3, 12%), IGF-1 (3, 6%) and leptin (4, 5%). Samples underwent a single freeze-thaw cycle.

### Statistical Analysis

Infant weight z-scores, fat mass and fat-free mass at discharge and 4 months' corrected age and weight gain between birth and 4 months' corrected age were considered as outcome variables as they are considered to represent the most important predictors for later metabolic health (39, 40). Waist and mid-upper-arm circumference at 4 months were used as a further indirect measure of body composition (41). MOM concentrations of leptin, adiponectin and IGF-1 were corrected for total protein concentration in the samples (mg/ml) and reported as ng/mg of protein per ml (ng/mg). Data on infant outcomes were tested for normality using the Shapiro-Wilk test. All outcomes were normally distributed with the exception of infant fat mass at discharge from hospital which was log_10_ transformed. Associations between MOM composition at each collection time-point and infant outcomes (weight z-score, fat mass and fat-free mass (kg), waist circumference and mid-arm circumference (cm) at discharge and 4 months' corrected age, weight gain between birth and 4 months' corrected age) were first assessed using unadjusted linear regression. The presence of associations between MOM composition at each time-point and the infant outcomes were then analyzed using multiple linear regression models. In this instance, each model was corrected for birthweight, total amount of MOM received during hospital admission, ml/day of formula milk consumed by the infant around the time of the follow-up visit (for all models assessing the 4-month outcomes) and GDM (only for models with discharge fat mass as outcome variable) as these variables were significantly correlated with the outcome variables in simple regression models (Supplementary Table 1). Analyses investigating the associations between MOM composition during early lactation and infant growth outcomes at discharge from hospital were inclusive of all infants whose mothers had given a MOM sample on day 5 and/or day 10 after birth. Analyses investigating associations between MOM composition during early lactation and infant outcomes at 4 months' corrected age were also inclusive of infants who were not breastfed at the time of follow-up whose mothers had given a MOM sample on day 5 and/or day 10 after birth. Analyses investigating associations between MOM composition at 4 months and infant outcomes at 4 months were inclusive only of infants who were still breastfed at 4 months and whose mothers had provided a sample at 4 months. Sex-specific interactions were also investigated in each model. All statistical analyses were performed using IBM SPSS (version 25) and graphs generated using Graph Pad Prism 8. Statistical significance was set at *p* < 0.05. All *p*-values shown in the context of sex-specific interactions refer to interaction effects. For this study, the power calculation was done a-posteriori. The power provided by the sample size of the smallest subgroup of the population (i.e., 4 months MOM composition as a determinant of 4 month infant outcome, *N* = 65) for simple and multiple linear regression and repeated-measure analysis calculated with G^*^Power 3.1.9.2 (University of Düsseldorf) as >85% at the 5% significance level for the detection of 20% difference across groups for all the measured main effects, while the power provided by the larger subgroups of the population provided a statistical power above 90% at the 5% significance level for the detection of 10% difference across groups for all the measured main effects.

## Results

### Population Characteristics

**Figure 1:**
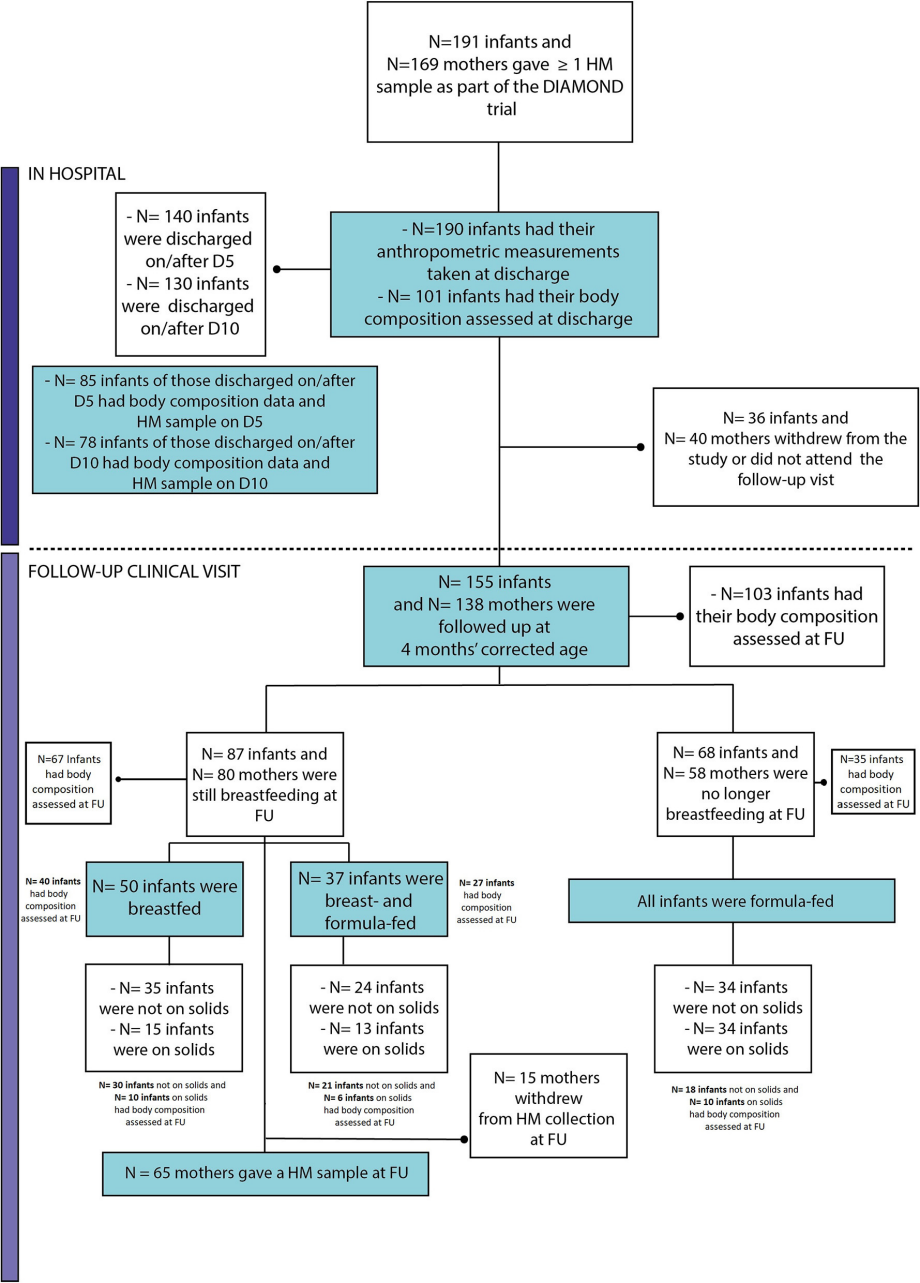
Flow chart of follow-up retention and infant feeding practices at 4 months' corrected age. FU, Follow-up; D5, day 5 (+2); D10, day 10 (±2).

Figure 1 summarizes the retention at discharge (99.5%) and follow-up (81%) and the infant feeding practice at 4 months' corrected age. The characteristics of this population have been described previously (13). A summary of the infant growth outcomes is shown in Table 1.

**Table 1:** Infant anthropometry and body composition across time from birth to 4 months' corrected age.

	**Birth**	**Discharge**	**4 months'**
		**from**	**corrected**
		**Hospital**	**age**
**Infant anthropometry (Mean, SD)**	**(*****N*** **= 191)**	**(*****N*** **= 190)**	**(*****N*** **= 155)**
Weight (g)	2,104 (417)	2,519 (331)	6,600 (862)
Weight (z-scores)	−0.1 (0.9)	−0.8 (0.8)	−0.1 (1.0)
Length (cm)	44.5 (3.0)	47.5 (2.0)	63.8 (2.5)
Length (z-scores)	0.2 (1.1)	−0.1 (0.9)	0.6 (1.1)
Head circumference (cm)	31.2 (1.5)	33.0 (1.2)	41.7 (1.4)
Head circumference (z-scores)	0.3 (0.9)	0.0 (0.7)	0.6 (1.0)
Waist circumference (cm)	–	30.4 (2.4)	40.8 (3.0)
Mid-upper-arm circumference (cm)	–	–	14.2 (1.3)
**Body composition (Mean SD)**		**(*****N*** **= 101)**	**(*****N*** **= 103)**
Fat mass (%)	–	10.1 (4.1)	25.7 (4.8)
Fat mass (kg)	–	0.3 (0.2)	1.7 (0.4)
Fat-Free mass (%)	–	89.9 (4.1)	74.3 (4.8)
Fat-Free mass (kg)	–	2.2 (0.2)	4.8 (0.5)
**Infant sex (*****N*****, %)**			
Female	81 (42.4)	81 (42.6)	65 (41.9)
Male	110 (57.6)	109 (57.4)	90 (58.1)

### MOM Composition and Infant Postnatal Growth Outcome

#### Infant Body Composition at Discharge From Hospital

For infants discharged on/after day 5 after birth, higher MOM protein concentration on day 5 after birth was associated with lower fat-free mass at discharge (Table 2). High protein in MOM were considered a proxy for higher formula milk at discharge (13) so we adjusted this model for the type of milk received by the infant at discharge but this did not alter our finding. Higher MOM leptin concentration on day 5 was associated with higher fat mass at discharge. Associations with MOM adiponectin and infant fat mass were found (*p* < 0.05) but were no longer statistically significant when the model was corrected for maternal GDM (Table 2), which was a strong predictor of infant fat mass. No other association was found between body composition at discharge from hospital and MOM composition at day 5 or day 10 after birth.

**Table 2:** Correlation between MOM composition and infant outcomes at discharge and 4 months' corrected age.

	**Discharge**								**Δ birth-4months' corrected age**	
	**Weight z-scores**		**Fat-Free mass (kg)**		**Fat mass [log**_****10****_**(kg)]**		**Waist circumference (cm)**		**Weight gain (g)**	
	**B (95% CI)**	***p***	**B (95% CI)**	***p***	**B (95% CI)**	***p***	**B (95% CI)**	***p***	**B (95% CI)**	***p***
**MOM composition on day 5**										
Leptin	0.03 (−0.96, 1.02)	0.947	0 (−0.28, 0.22)	0.972	**0.33 (0.07, 0.59)**	**0.013**	0.30 (−2.08, 2.67)	0.807	−218 (−1,098, 661)	0.626
Adiponectin	0.03 (−0.14, 0.19)	0.744	0.04 (0.01, 0.07)	0.014	−0.05 (−0.17, 0.06)	0.359	−0.08 (−0.97, 0.81)	0.857	−24 (−164, 116)	0.735
IGF-1	0.57 (−3.36, 4.51)	0.775	0.64 (−0.93, 2.20)	0.427	1.18 (−0.43, 2.78)	0.15	17.11 (−9.33, 43.56)	0.205	3,384 (−1,957, 8,724)	0.214
Protein	−0.01 (−0.04, 0.02)	0.533	**−0.02 (−0.03, 0)**	**0.029**	−0.01 (−0.03, 0.01)	0.187	−0.09 (−0.26, 0.07)	0.264	−21 (−59, 17)	0.285
**MOM composition on day 10**										
Leptin	1.13 (−1.01, 3.28)	0.301	1.34 (−0.01, 2.70)	0.051	1.27 (−0.27, 2.80)	0.105	7.96 (−7.88, 23.79)	0.325	1,129 (−3,473, 5,731)	0.631
Adiponectin	0.03 (−0.11, 0.17)	0.686	0.03 (−0.01, 0.07)	0.111	−0.01 (−0.08, 0.05)	0.74	0.17 (−0.46, 0.80)	0.592	−49 (−176, 77)	0.444
IGF-1	0.32 (−1.52, 2.16)	0.735	0.18 (−0.75, 1.12)	0.701	0.20 (−1.21, 1.62)	0.78	9.49 (−3.97, 22.95)	0.167	333 (−1,201, 1,866)	0.67
Protein	0.02 (−0.02,0.05)	0.299	−0.01 (−0.02,0.01)	0.211	−0.01 (−0.03, 0.01)	0.454	−0.07 (−0.22, 0.07)	0.339	18 (−39, 75)	0.539
**MOM composition at 4 months**										
Leptin	–	–	–	–	–	–	–	–	−81 (−2,636, 2,473)	0.95
Adiponectin	–	–	–	–	–	–	–	–	−210 (−462, 42)	0.103
IGF-1	–	–	–	–	–	–	–	–	249 (−1,275, 1,774)	0.748
Protein	–	–	–	–	–	–	–	–	−24 (−52, 4)	0.099
	**4 months' corrected age**									
	**Weight Z**		**Fat-free mass (kg)**		**Fat mass (kg)**		**Waist circumference (cm)**		**Mid-Upper-Arm circumference (cm)**	
	**B (95% Cis)**	***p***	**B (95% Cis)**	***p***	**B (95% Cis)**	***p***	**B (95% Cis)**	***p***	**B (95% Cis)**	***p***
**MOM composition on day 5**										
Leptin	−0.24 (−1.41, 0.93)	0.686	0.54 (−0.17, 1.24)	0.134	**−0.72 (−1.33**, **−0.11)**	**0.021**	**−4.44 (−7.76**, **−1.12)**	**0.009**	**−2.78 (−4.25**, **−1.31)**	** <0.001**
Adiponectin	−0.01 (−0.18, 0.17)	0.949	0.02 (−0.05, 0.10)	0.566	−0.01 (−0.08, 0.06)	0.739	0.02 (−0.47, 0.52)	0.923	−0.11 (−0.39, 0.17)	0.434
IGF-1	4.71 (−2.00, 11.41)	0.169	**5.23 (1.38, 9.08)**	**0.008**	1.70 (−3.56, 6.96)	0.526	1.84 (−23.24, 26.92)	0.886	1.99 (−9.93, 13.92)	0.743
Protein	−0.03 (−0.08, 0.02)	0.226	**−0.04 (−0.06**, **−0.02)**	**0.002**	−0.01 (−0.04, 0.01)	0.327	−0.01 (−0.19, 0.02)	0.884	−0.03 (−0.11, 0.05)	0.459
**MOM composition on day 10**										
Leptin	0.74 (−5.81, 7.30)	0.824	1.06 (−1.53, 3.65)	0.423	0.08 (−3.54, 3.69)	0.967	2.79 (−13.03, 18.60)	0.73	3.44 (−5.15, 12.03)	0.432
Adiponectin	−0.04 (−0.21, 0.12)	0.611	0.01 (−0.05, 0.07)	0.644	−0.05 (−0.11, 0.01)	0.072	0.02 (−0.35, 0.39)	0.915	−0.12 (−0.39, 0.15)	0.384
IGF-1	0.05 (−2.00, 2.10)	0.963	0.65 (−0.36, 1.66)	0.21	0.63 (−0.45, 1.70)	0.253	4.16 (−3.16, 11.47)	0.265	2.42 (−1.44, 6.28)	0.219
Protein	0.02 (−0.05, 0.08)	0.64	−0.01 (−0.05, 0.04)	0.824	0.01 (−0.03, 0.05)	0.489	0.10 (−0.14, 0.34)	0.411	−0.01 (−0.10, 0.08)	0.869
**MOM composition at 4 months**										
Leptin	0.01 (−3.15, 3.17)	0.955	0.49 (−2.31, 3.28)	0.733	−1.26 (−3.24, 0.72)	0.212	−1.73 (−9.99, 6.53)	0.682	−3.25 (−7.52, 1.01)	0.135
Adiponectin	−0.23 (−0.56, 0.10)	0.177	0.03 (−0.21, 0.27)	0.778	−0.08 (−0.22, 0.05)	0.219	0.39 (−0.90, 1.68)	0.554	−0.21 (−0.83, 0.41)	0.506
IGF-1	0.47 (−1.61, 2.55)	0.656	0.31 (−0.63, 1.25)	0.513	0.12 (−0.60, 0.83)	0.75	**7.47 (3.98, 10.97)**	** <0.001**	0.20 (−1.74, 2.13)	0.844
Protein	−0.03 (−0.08, 0.01)	0.137	−0.01 (−0.02, 0.01)	0.369	−0.02 (−0.05, 0)	0.056	−0.14 (−0.31, 0.03)	0.099	−0.08 (−0.15, 0)	0.057

*The values shown in the table are based on the fitted multiple linear regression models. All models were corrected for Total MOM received during hospital admission via enteral tube and birthweight. Models with discharge fat mass as outcome were corrected for GDM and all models involving a 4 month outcome were corrected for ml/day of formula milk consumed by the infant at the time of follow-up*.

*IGF-1, Insulin-like growth factor-1*.

*Hormone concentrations are reported in ng/mg and protein concentration in mg/ml*.

*B, unstandardized beta coefficient. Beta coefficients represent the amount of change in the dependent variable (e.g., weight) per unit change in hormone (ng/mg). Bold values are statistically significant*.

#### Weight Gain and Body Composition at 4 Months' Corrected Age

There was no correlation at any time point between the MOM components analyzed in this study and infant weight gain from birth to follow-up (Table 2). Higher MOM protein concentration on day 5 was associated with lower fat-free mass at 4 months, while higher MOM IGF-1 on day 5 was associated with higher fat mass at 4 months (Table 2). In contrast to that observed at discharge from hospital, higher concentrations of leptin in MOM on day 5 were associated with lower fat mass, waist and mid-upper-arm circumference at 4 months' corrected age (Table 2). Higher MOM IGF-1 at 4 months' corrected age was associated with higher waist circumference at 4 months (Table 2). Associations with MOM adiponectin and infant fat mass was again found at 4 months' corrected age (p < 0.05); however, once more, this association was no longer statistically significant when the model was corrected for maternal GDM (Table 2), which was a strong predictor of infant fat mass.

#### Sex-Specific Growth Outcomes in Relation to MOM Composition

**Figure 2:**
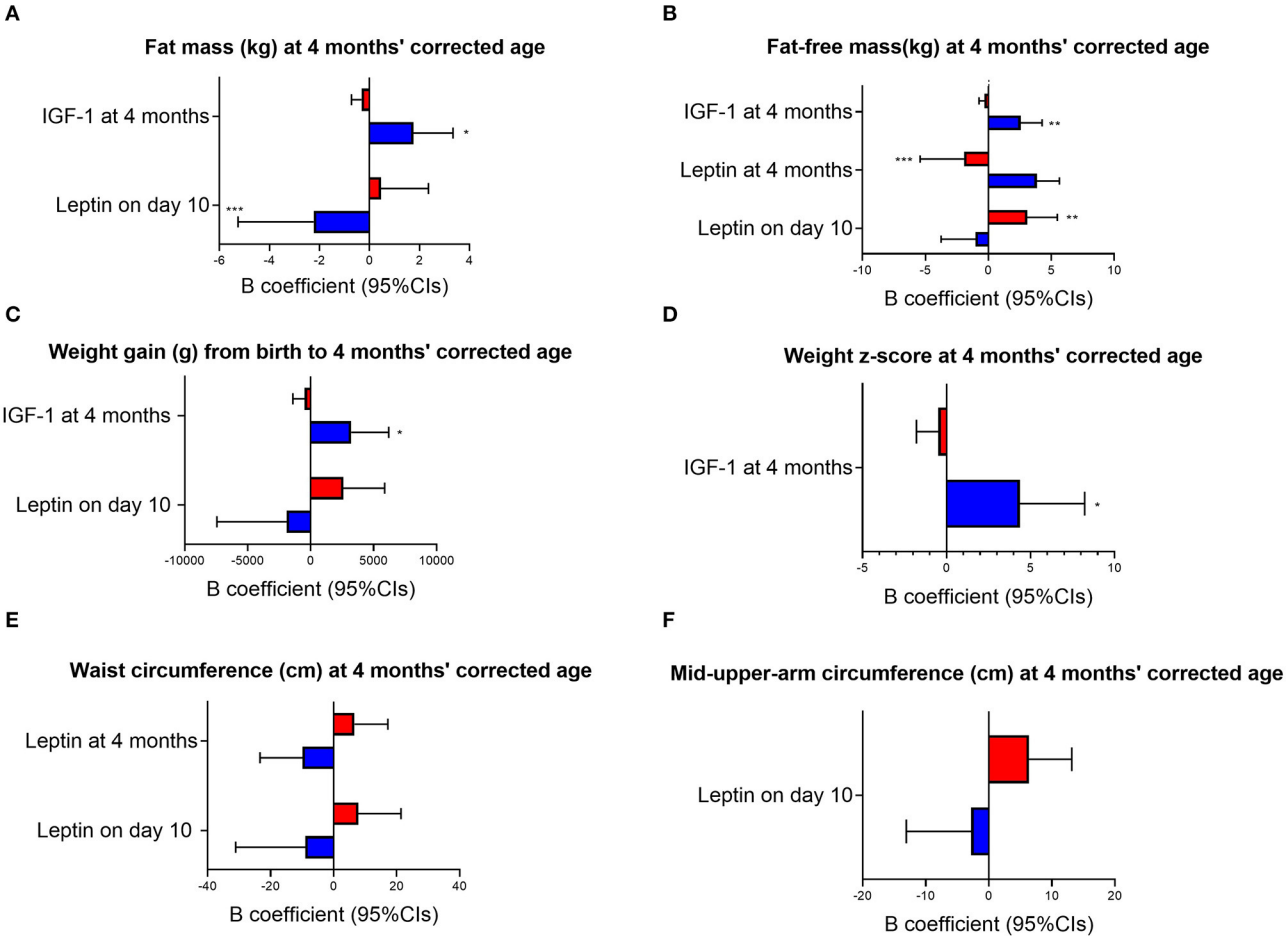
Sex-specific response to MOM composition. Red bars, females; blue bars, males; IGF-1, Insulin-like growth factor-1; Cis, Confidence Intervals. The graphs are based on multiple linear regression where birthweight, ml/day of formula consumed by the infant at 4 months' corrected age and total amount of MOM received in hospital via enteral tube were used as covariates. Concentrations of all HM compounds are reported in ng/mg. B, unstandardized beta coefficients. *P*-values specified in the text refer to interaction terms (i.e., sex*MOM compound). *p ≤ 0.05, **p ≤ 0.01, ***p ≤ 0.001 in subgroups analyses (i.e., only males and only females).

Sex-specific interactions between MOM composition and infant growth outcomes were observed in the cohort (Figure 2). Overall, higher MOM IGF-1 at 4 months' corrected age was associated with increased growth outcomes in males and a reduction or no difference in female growth. Specifically, male and female infants showed different trajectories to MOM components in terms of fat mass (males showing reduced fat mass in association with an increase in leptin *p* = 0.003 for leptin concentrations on day 10 and *p* = 0.022 for IGF-1 concentrations at 4 months, Figure 2A), fat-free mass (*p* = 0.007 for leptin on day 10, *p* = 0.004 for leptin at 4 months and *p* = 0.001 for IGF-1 at 4 months, Figure 2B), weight z-score (*p* = 0.014 for IGF-1 at 4 months, Figure 2D), waist circumference (*p* = 0.045 for leptin on day 10 and *p* = 0.033 for leptin at 4 months, Figure 2E) and mid-upper-arm circumference (*p* = 0.021 for leptin on day 10, Figure 2F) at 4 months' corrected age, and weight gain from birth to 4 months (*p* = 0.026 for leptin on day 10 and *p* = 0.016 for IGF-1 at 4 months, Figure 2C).

## Discussion

We investigated associations between the concentrations of MOM metabolic hormones at different times during lactation and growth outcomes in a moderate-late preterm cohort at discharge from hospital and at 4 months' corrected age. The results confirmed our hypothesis that variability in MOM hormonal composition may contribute to variation in postnatal growth and that infants of different sex are likely to respond differently to the same concentrations of MOM components. Importantly, the data also confirm our previous observations in the healthy Finnish STEPS cohort regarding the correlations between MOM IGF-1 concentrations and infant weight (18), highlighting the possibility that exposure to varied MOM IGF-1 concentrations is associated with variation in infant growth outcomes (e.g., weight, BMI, body composition).

We observed fat-free mass at discharge from hospital and at 4 months' corrected age was lower for infants whose mothers had a higher MOM protein content during early lactation (day 5 postpartum). This is in contrast to studies in very low birth weight infants which show that increased protein intakes are beneficial for lean mass growth (42), but is in accordance with scientific evidence showing that the intake of high protein formula in the first year of life is linked to increased body fat (43, 44). It could therefore be speculated that high intakes of protein in early life may indeed not be optimal for lean mass growth, and suggest that alterations increasing the concentration of protein in MOM might predispose the infant to develop a body composition with less fat-free mass. Interestingly, in our previous analyses, we found that, non-elective C-section and GDM were associated with increased MOM protein concentrations only in MOM produced for sons (13). This suggests that there are specific environmental conditions which may trigger either physiological or pathological responses that alter the compositional profile of MOM, often in a sex-specific manner, and that might ultimately have a role in directing infant growth trajectories also in the postnatal period. Whether these responses are part of evolutionary mechanisms with a meaning in the ecology of our species is unknown. However, while this area of research is currently very poorly represented, especially in clinical settings, understanding the etiology of such responses is essential if we want to give the best start to the lives of all babies.

MOM leptin concentration was also associated with infant body composition at discharge. Despite leptin being one of the most studied bioactive compounds in MOM, its relationship with infant outcomes and body composition is still a point of debate. Previous studies have found associations between higher MOM leptin and increased infant fat mass (20), and some speculate that leptin may be one of the key factors in maternal-offspring obesity transmission (45). Data across a range of experimental animal models has highlighted that maintenance of a critical leptin level during early life plays a key role in reducing obesity risk in later life (46). However, data in the human are conflicting. Numerous studies to date have provided clear evidence that HM leptin is positively correlated with maternal BMI (47) although some have dismissed the link between leptin and adiposity following observations that HM leptin concentrations were not different between mothers of obese and non-obese infants (48). In our study, we found that higher MOM leptin concentrations during early lactation were associated with higher fat mass at discharge from hospital but with decreased fat mass, waist circumference and mid-upper-arm circumference at 4 months' corrected age. This aligns with previous observations by Fields et al. (49), Fields and Demerath (50), where leptin was shown to be associated with increased weight gain from 0 to 1 month of age but negatively associated with fat mass at 6 months of age. However, given that the present study lacked information on pre-pregnancy BMI, a known predictor of HM leptin and fat mass in infancy, it is difficult to directly determine the implications of the results observed in the present study without controlling for such a maternal factor. Hence, together with the current available literature detailing conflicting associations between HM leptin and infant growth trajectories, our findings cannot secure any definitive conclusions around this linkage. As previous studies have already noted (51), accounting for the interactions that leptin has with other hormones, including glucocorticoids (52) and other metabolic hormones (53, 54) might be necessary to fully understand the role of leptin in relation to infant growth trajectories. Additionally, taking into consideration the possibility that HM leptin may signal maternal nutritional and metabolic status and perhaps the food availability in the environment the infant will face after weaning might also be important in order to fully understand the programming role of this metabolic hormone.

As regards adiponectin, although previous studies have reported associations between HM adiponectin and infant growth (55), the available evidence to date is also conflicting, with other studies reporting no association between HM adiponectin and infant growth (19). In the present study in a preterm cohort we did observe that MOM adiponectin was associated with lower infant fat mass at discharge and at 4 months' corrected age. However, this association was no longer significant once the model was corrected for maternal GDM, which generally, and in our cohort, was a strong predictor of infant fat mass. Both in this cohort and in the Finnish STEPS cohort we found that GDM was also associated with lower MOM adiponectin (4, 13). This, together with other existing evidence, suggests that GDM might alter HM composition (56–58). Therefore, while previous studies have reported on the association between GDM exposure *in utero* and infant fat mass (59), it is also possible that GDM might alter the composition of HM throughout lactation and may in part be responsible for shifting the body composition of the infant toward a higher fat mass phenotype. However, at present there remains a lack of studies investigating a possible role of HM composition in mediating the detrimental effects of GDM in relation to infant body composition and long-term infant health outcomes.

The present study confirms our previous findings in the Finnish STEPS cohort (18) that there is a significant association between IGF-1 and infant growth. Higher MOM IGF-1 concentrations during early lactation were associated with an increase in fat-free mass but not with an increase in weight gain. Higher MOM IGF-1 concentrations at 4 months' corrected age were instead associated with a greater waist circumference. Previous studies around circulating IGF-1 during infancy confirm the relationship between IGF-1 and leptin concentrations and the association of both these compounds with infant fat and, to a lesser extent, lean mass (60–62). Previous studies have reported on a strong correlation between waist circumference and fat mass (63) although in our cohort IGF-1 did not show any association with fat mass. Preterm infants have been reported to have altered circulating IGF-1 and consequent developmental issues and impairment of nutrient utilization (64). IGF-1 supplementation has been proposed to improve these conditions. However, there remain a lack of data around the possible impact of IGF-1 on preterm infants in the long term (65, 66). Similar to our previous findings in the STEPS Finnish cohort, the results of the present study seem to suggest that increased IGF-1 in HM, and specifically during early lactation, may favor a beneficial effect in relation to body composition during early life and better metabolic outcomes in the longer term (e.g., lower BMI, lower weight gain, increased fat-free mass). In terms of evolutionary pressures, researchers have suggested that individuals whose mothers can afford to invest more in their offspring during pregnancy and lactation typically have a healthier body composition, better health outcomes and a longer lifespan (12). In this context, considering that circulating IGF-1 is involved in lean mass and bone accretion (67), and is strictly related to the intake of nutrients that favor the accretion of bone and lean mass (68), the concentration of IGF-1 in HM might constitute an environmental cue for the offspring. In particular it could be that, depending on the amount of HM-borne IGF-1 received during lactation, the offspring might be programmed toward the growth trajectory that would be more favorable under the anticipated environment they will face once weaned, which is the same environment that has determined the composition of the HM they are receiving. We have previously reported that maternal pre-pregnancy BMI, weight gain during pregnancy, delivery mode and socioeconomic status were all associated with IGF-1 concentrations in MOM. In both this moderate-late preterm cohort and the Finnish STEPS cohort (4) we found sex-specific associations between MOM IGF-1 concentrations and socioeconomic status, with mothers living in good neighborhoods producing HM with similar quantity of IGF-1 for male and female infants but mothers living in lower socioeconomic status producing HM with higher IGF-1 for males. Considering the importance of understanding the early determinants of postnatal outcomes, future studies should investigate, in a more comprehensive manner, the role of maternal environment (socioeconomic status and health) on HM hormonal profile and on subsequent growth outcomes.

Sex-specific effects on growth outcomes were observed in relation to MOM composition and represent a key finding of this study. Sex-specific responses in terms of growth outcomes at 4 months' corrected age were also observed in relation to MOM IGF-1 and leptin both during early and late lactation, with male and female infants displaying different growth patterns, and males in particular showing increased growth outcomes when exposed to MOM with more abundant IGF-1 content at 4 months' corrected age. Sex-specific responses in terms of increased risk for cardio-metabolic disorders under different maternal conditions and dietary exposures during lactation (69, 70) and in relation to growth trajectories to hormonal exposures through milk during lactation (71) have also been reported in animal models including mice, rats and primates. However, to our knowledge, no previous study has investigated the association between hormonal exposures through lactation and sex-specific responses in humans.

One of the main limitations of the present study was the lack of data around maternal pre-pregnancy BMI and other maternal information (e.g., smoking, dietary habits). These can be primary predictors for HM composition and infant birth-weight and growth. Furthermore, the trial did not collect maternal, infant or cord blood, which would have been valuable for comparing the associations between circulating and HM concentrations and potential mechanisms of action related to our observations. In the present study, it was difficult to accurately control for the dietary intakes of the infant as the population had variable nutritional patterns throughout the duration of the study. While this could have been controlled for if dietary information was collected from birth to follow-up, this was not done in the present study. However, such additional data would have allowed for a more accurate correction of the statistical models and hence it is recommended that nutritional intakes are recorded throughout the study duration in future studies evaluating associations between HM composition and infant growth outcomes/body composition. Due to the nature of the study population, the groups presented in this study were not perfectly balanced. However, we have controlled our statistical analyses for all the factors that significantly correlated with the outcome variables. Further, for the present study, we have not corrected our statistical models for factors that affect HM composition in order to evaluate associations between HM variability (associated with maternal SES and health factors) and infant growth. Nonetheless, this study had considerable strengths, including the collection of HM at multiple time-points over lactation, the collection of body composition data together with the infant anthropometry, the cohort sample size and the availability of a number of other parameters (i.e., amount of HM received in hospital and amount of formula consumed by the infant at 4 months' corrected age) that allowed us to improve as best as possible our fitted statistical models.

Overall, the present study confirmed our hypothesis and added further evidence to the body of literature that suggests that HM composition throughout lactation possibly influences infant growth or mediates maternal conditions, such as GDM, that are known to have an effect on infant growth. Changes in HM IGF-1 in particular seem to have a role in defining infant body composition and may be important in mediating the effect of environmental stimuli on developmental programming processes that happens during early life. Our results show that the response to HM concentrations of IGF-1 and leptin may vary depending upon the sex of the infant, with males potentially being more affected by a variation in the composition of the HM they receive, as opposed to females. This might suggest that female and male infants have different requirements to thrive. Furthermore, as HM-borne hormones might function as chemical messengers from mother to infant, and hormonal concentration in HM appear to be linked to maternal health and socioeconomic environment, the present study together with our previous research seems to suggest that the support provided to mothers (e.g., counseling, nutrition advice) may need to be tailored for each mother-infant dyad based on individual needs. Ensuring opportunities are in place for all mothers to produce the best HM they can may translate into improved postnatal outcomes for all infants. Further studies are required to better understand (1) the effect that improved and tailored support to mothers has on MOM composition and (2) the extent of sex-specific responses to early life nutrition. If confirmed, our observations would have an important impact on current clinical practices and policies for maternal support and infant feeding, as well as a pivotal role in the understanding of the origin of sex-specific diseases and morbidity risk.

## Data Availability Statement

The raw data supporting the conclusions of this article will be made available by the authors, without undue reservation.

## Ethics Statement

The study protocol was approved by the New Zealand Health and Disability Ethics Committee (16/NTA/90). The patients/participants provided their written informed consent to participate in this study.

## Author Contributions

LG drafted the collection protocol for human milk samples, assisted with sample and data collection, performed laboratory work, data analysis and result interpretation, and wrote the first draft of the manuscript. CR and AM provided input in the design of the collection and laboratory protocols. FB and TA designed the randomized controlled trial, provided funding for sample and data collection, and together with DC-S contributed to the collection protocol. MV provided funding for sample analysis and together with SP provided oversight of the process from analysis through to manuscript submission. All authors critically reviewed and edited the manuscript and approved the final version.

### Conflict of Interest

AM was employed by the company AgResearch Limited. The remaining authors declare that the research was conducted in the absence of any commercial or financial relationships that could be construed as a potential conflict of interest.
