# Supplementary material for: Metabolic Hormone Profiles in Breast Milk From Mothers of Moderate-Late Preterm Infants Are Associated With Growth From Birth to 4 Months in a Sex-Specific Manner
Source: Front Nutr. 2021 May 28;8:641227. doi: 10.3389/fnut.2021.641227 (PMC8193224; doi:10.3389/fnut.2021.641227)
Supplement: Supplementary file 1 [file Table_1.pdf]

Supplementary Table 1. Factors used to correct the fitted statistical models.

Discharge group	N (%)
GDM*	36 (19)
no GDM*	154 (81)
**Nutrition received**	**Mean (SD)**
Total MOM consumed in hospital (ml)	2057 (172)

4M group	N(%)
GDM*	28 (18)
no GDM*	127 (82)
**Nutrition received**	**Mean (SD)**
Total MOM consumed in hospital (ml)	2150 (193)
Formula milk received at 4M (ml/day)	560 (5)

*only models with discharge fat mass as outcome variable were corrected for GDM, as GDM significantly associated with infant fat-mass
